# Statins and Hip Fracture Prevention – A Population Based Cohort Study in Women

**DOI:** 10.1371/journal.pone.0048095

**Published:** 2012-10-29

**Authors:** Arja Helin-Salmivaara, Maarit J. Korhonen, Petri Lehenkari, Seppo Y. T. Junnila, Pertti J. Neuvonen, Päivi Ruokoniemi, Risto Huupponen

**Affiliations:** 1 Department of Pharmacology, Drug Development and Therapeutics, University of Turku, Turku, Finland; 2 Unit of Primary Health Care, Hospital District of Helsinki and Uusimaa, Helsinki, Finland; 3 Department of Anatomy and Cell Biology, University of Oulu, Oulu, Finland; 4 Division of Surgery, Department of Surgery and Intensive Care, Clinical Research Centre, Institute of Clinical Medicine, University of Oulu, Oulu University Hospital, Oulu, Finland; 5 Salo Health Care Centre, Salo, Finland; 6 Department of Clinical Pharmacology, University of Helsinki, Helsinki, Finland; 7 Unit of Clinical Pharmacology, Turku University Hospital, Turku, Finland; University of Milan, Italy

## Abstract

**Objective:**

To study the association of long-term statin use and the risk of low-energy hip fractures in middle-aged and elderly women.

**Design:**

A register-based cohort study.

**Setting:**

Finland.

**Participants:**

Women aged 45–75 years initiating statin therapy between 1996 and 2001 with adherence to statins ≥80% during the subsequent five years (n = 40 254), a respective cohort initiating hypertension drugs (n = 41 610), and women randomly selected from the population (n = 62 585).

**Main Outcome Measures:**

Incidence rate of and hazard ratio (HR) for low-energy hip fracture during the follow-up extending up to 7 years after the 5-year exposure period.

**Results:**

Altogether 199 low-energy hip fractures occurred during the 135 330 person-years (py) of follow-up in the statin cohort, giving an incidence rate of 1.5 hip fractures per 1000 py. In the hypertension and the population cohorts, the rates were 2.0 per 1000 py (312 fractures per 157 090 py) and 1.0 per 1000 py (212 fractures per 216 329 py), respectively. Adjusting for a propensity score and individual variables strongly predicting the outcome, good adherence to statins for five years was associated with a 29% decreased risk (HR 0.71; 95% CI 0.58–0.86) of a low-energy hip fracture in comparison with adherent use of hypertension drugs. The association was of the same magnitude when comparing the statin users with the population cohort, the HR being 0.69 (0.55–0.87). When women with poor (<40%), moderate (40 to 80%), and good adherence (≥80%) to statins were compared to those with good adherence to hypertension drugs (≥80%) or to the population cohort, the protective effect associated with statin use attenuated with the decreasing level of adherence.

**Conclusions:**

5-year exposure to statins is associated with a reduced risk of low-energy hip fracture in women aged 50–80 years without prior hospitalizations for fractures.

## Introduction

Impact of statins (hydroxymethylglutaryl-CoA reductase inhibitors) on bone mineral density (BMD) has been debated since simvastatin and lovastatin were discovered to increase bone formation in animal experiments [1]. At the cellular level, there is no doubt that statins can affect bone formation. By inhibiting mevalonate pathway, statins decrease protein isoprenylation with a subsequent activation of bone morphogenetic protein-2 which contributes to osteoblast differentiation [1], [2]. Further support for a biological effect of statins comes from a recent experimental study which showed a drastic beneficial effect of locally administered simvastatin on fracture healing [3].

In clinical studies, observations on the effects of statins on bone have been inconsistent, especially in postmenopausal women, the most vulnerable population in terms of low energy fractures and bone health. While one meta-analysis showed the effect of statin use on various bone turn over markers in postmenopausal women [4], another more recent meta-analysis found no effect of statin use (1 year or less) on these markers in the same population [5]. A modest positive effect of statin use on hip BMD in women was found in two meta-analyses including various types of studies [4], [6]. This finding could not be verified, however, when only randomised trials were considered [5].

In theory, statins may have impact on both the BMD and micro architecture and molecular composition of bone. Overall, the role of BMD as a sole predictor of fractures has been questioned since only a small proportion of patients with a low-energy fracture have a decreased BMD [7]. Besides it, the micro architecture and molecular composition of the bone may play a significant role determining the bone quality [8].

Concerning hip fracture in women, a typical fracture in osteoporosis, statin use was associated with a 25% reduced risk (odds ratio [OR] 0.75; 95% CI 0.60–0.95) in a meta-analysis of nine studies [4]. When considering any fracture in women, the OR for a fracture associated with statin use was 0.80 in a meta-analysis of 11 studies (0.66–0.96) [9]. The risk of any fracture in women was analysed in two post-hoc studies of randomised trials on cardiovascular end points [10], [11]. In the AFCAPS/TexCAPS study, only one hip fracture occurred in each (lovastatin and placebo) group [10]. In the LIPID study, data on the risk of hip fracture in women was not presented [11] but the risk ratio for any fracture favoring pravastatin was 0.78 (95% CI 0.54–1.14) [5], [11].

We hypothesised that the exposure to statins should be at least as long as bone renewal time in order to demonstrate an association between statin use and such clinical outcomes as low-energy osteoporotic fractures. As the exact duration of bone renewal in hip in middle-aged or elderly women is not known, we arbitrarily set the minimum exposure time to five years. In several observational studies on statin use and risk of hip fracture in women, the exposure periods have been considerably shorter than five years [12], [13], [14]. Furthermore, in four prospective studies with a similar follow-up time, the analyses were intention-to-treat, the exposure being defined at baseline [15].

We performed a register-based study on the association of long-term statin use with the risk of low-energy, potentially osteoporotic fractures in middle-aged and elderly women. The primary aim was to compare the incidence of low-energy hip fracture between adherent users of statins and adherent users of antihypertensive drugs. The comparator group was selected in order to control for health seeking behaviour [16] and risk factors for cardiovascular diseases and fractures unavailable in the registers. Secondly, the incidence of low-energy hip fracture between statin users and a randomly selected population cohort was compared.

## Methods

### Sources of Data

We used data from administrative health databases generated through the universal health care and drug reimbursement systems covering the 5.4 million residents of Finland. We identified prescription records with the Prescription Register run since 1994 and managed by the Social Insurance Institution (SII) [17]. This register contains records of prescription drug purchases reimbursed to residents in non-institutional settings. For each purchase, the data include the dispensing date, the Anatomical Therapeutic Chemical classification code presented by the WHO [18], and the quantity dispensed. Permanent residents of the country are eligible to drug reimbursement and are therefore included in the Register, even if they did not get any reimbursement. Patients staying in a public nursing home or hospital without interruption for over 90 days are not eligible for drug reimbursement, and their purchases are not registered. We identified these patients from a separate SII register. For identifying patients entitled to higher rates of reimbursement because of certain severe, chronic conditions, such as coronary artery disease, diabetes, rheumatoid arthritis, and organ transplantations, we used the SII Special Reimbursement Register introduced in 1964. To be eligible for special reimbursement, a patient’s condition must meet explicit predefined criteria, and a written certificate by a physician is required.

We identified low-energy hip fractures from the Finnish Care Register, managed by the National Institute of Health and Welfare. The register, covering all Finnish hospitals and all hospitalizations regardless of the payer, includes individual administrative data on primary and secondary discharge diagnoses and the admission and discharge dates. The 10th revision of the International Classification of Diseases (ICD-10) has been in use since January 1, 1996. Properties of the fall (place and reason) leading to a hospitalised injury can be registered but it is not compulsory. The validity of the Finnish Care Register for hip fractures has been tested comparing medical records, prospective audit data, and the corresponding register data for 106 consecutive patients hospitalised for hip fracture in one hospital in 1999–2000 [19]. The sensitivity of hip fracture diagnosis in the register varied from 68.3% to 96.7%, depending on the specific site of the fracture.

The data from the above databases were linked anonymously using encrypted personal identifiers.

### Cohorts and Exposure

**Figure 1:**
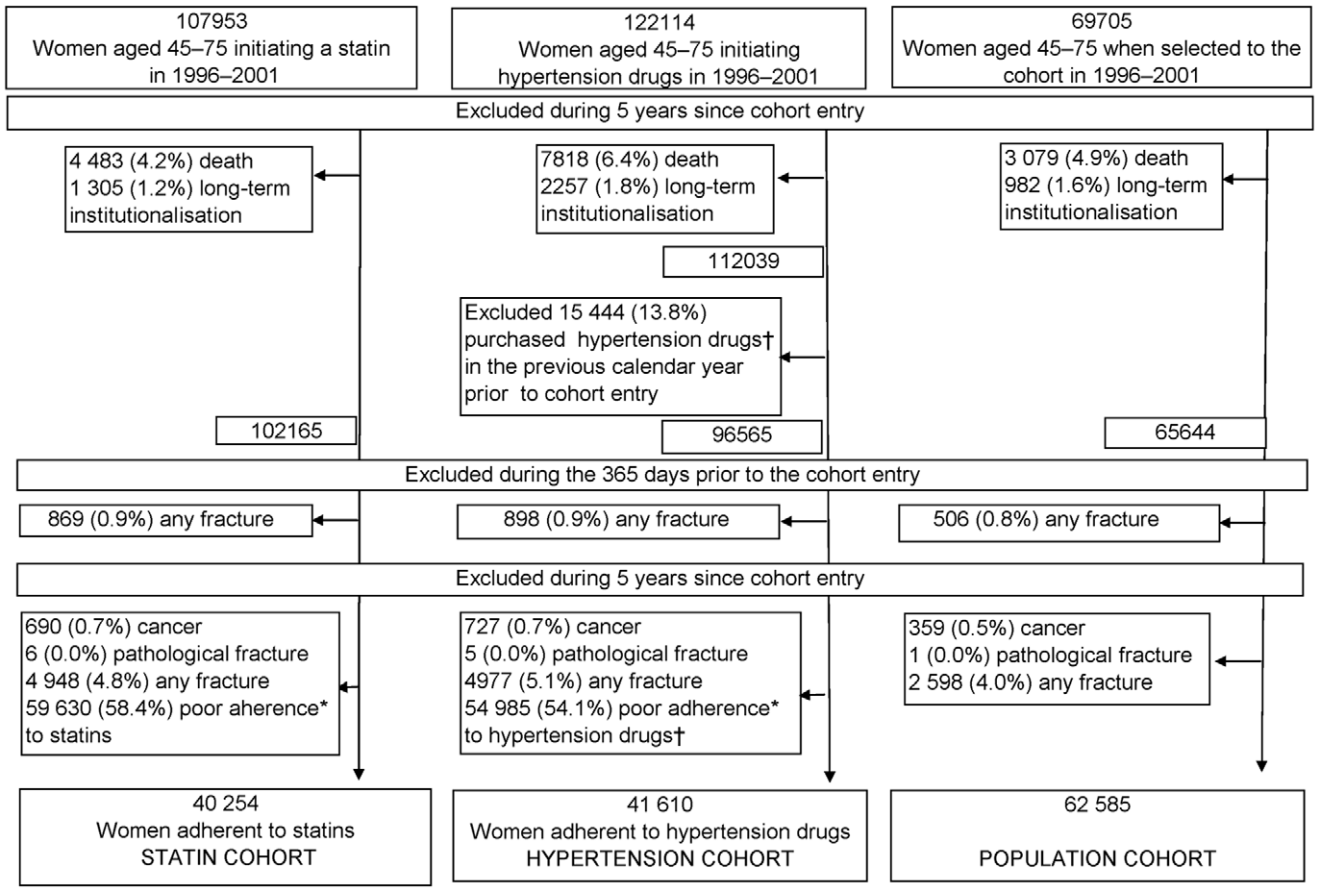
Flow diagram of the study cohorts. *Poor adherence = prescribed days covered <80% in the 5-year exposure period and no more than 2 purchased statin/hypertension drug prescription in each year. †Hypertension drugs = beta blockers, angiotensin-converting enzyme inhibitors or angiotensin receptor blockers, calcium channel blockers.

**Figure 2:**
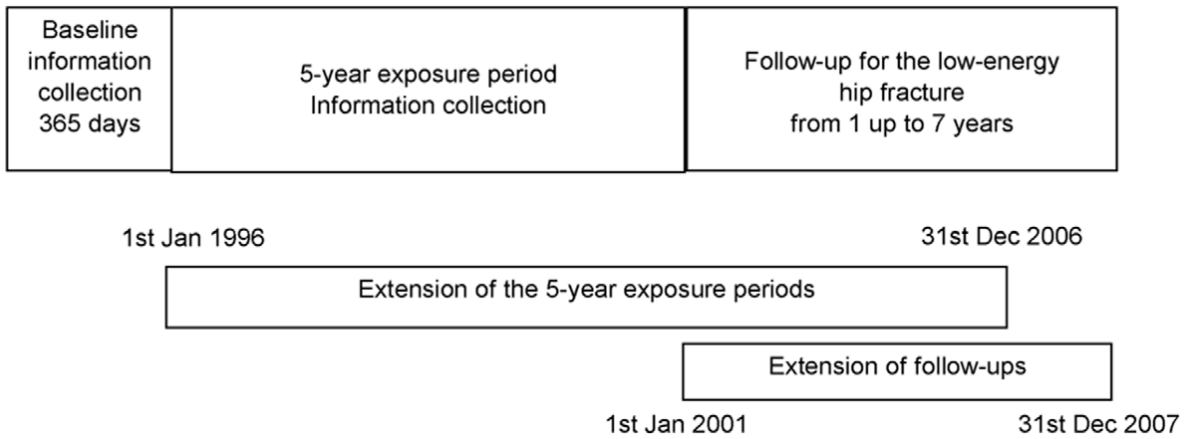
The study exposure and follow-up periods in relation to the calendar time. The figure is not to scale.

Women aged 45–75 years by the end of the calendar year prior to each selection year were eligible to be included in the source population (Figure 1). Persons not considered eligible were those entitled to special drug reimbursement due to organ transplantations, severe renal insufficiency, severe gastrointestinal disease (mostly inflammatory bowel diseases), or Alzheimeŕs disease between 1996 and 2007. We excluded persons who died or were institutionalised during the 5-year exposure period since the cohort entry. From the hypertension cohort, we excluded persons who had purchased antihypertensive drugs in the year preceding the cohort entry. Next, we excluded from all cohorts those persons who had had any fracture in the 365 days prior to the cohort entry and those who had been hospitalised with any cancer diagnosis (except for non-melanoma skin cancer), with a diagnosis of pathological fracture, or with any fracture during the 5-year exposure period. The follow-up for the cohorts started 5 years since the cohort entry and ended when a person sustained a low-energy hip fracture, met any of the exclusion criteria mentioned above or on Dec 31, 2007 whichever came first (Figure 2). When sampling the cohorts, we strove to similar distributions of the cohort entry years.

#### Statin cohort

The statin cohort consisted of new users of statins from 1996 to 2001. The cohort entry was defined as the date of the first statin purchase. Those who had purchased statins in 1994 or 1995 were excluded, and each person was considered as an incident user only once. For the main analyses, the cohort was restricted to those persons adherent to statins in the 5-year exposure period. Adherence was defined as the Proportion of Days Covered (PDC) ≥80% [20]. We used the one-tablet-a-day dosage assumption when calculating the PDC, i.e. a person had to have purchased at least 1460 statin tablets during the 5 years (1825 days). Furthermore, at least 3 statin dispensations each year were required. In Finland, a drug can be reimbursed for no longer than 3 months’ therapy per purchase. Statin use after the exposure period was not considered.

For exposure-response relationship analyses, all statin initiators were further divided into three groups of adherence in the 5-year period: <40%, 40–80%, and ≥80% plus at least 3 purchases each year.

#### Hypertension cohort

The hypertension cohort consisted of women who had not purchased drugs indicated in hypertension (imidazoline receptor agonists, beta blockers, angiotensin-converting enzyme inhibitors or angiotensin receptor blockers, or calcium channel blockers) any time since 1994 until their first antihypertensive drug purchase between 1996 and 2001. The date of the first purchase defined the cohort entry. As diuretics are indicated also for several other conditions their use was not considered in the cohort definition. We restricted the hypertension cohort to those adherent to antihypertensive drugs or diuretics in the 5-year exposure period using the same definition for the adherence as in the statin cohort. Switching between drugs was considered as continuing the therapy. Persons that purchased statins in 1996–2006 could not be included in the hypertension cohort.

#### Population cohort

The population cohort consisted of women randomly selected from the source population by using the Prescription Register of the SII. Those who had no statin purchases or who had no more than one statin purchase at any point in 1995–2006 were included. The cohort entry date was set at the 30^th^ of June of the selection year.

### Study End Points

We measured the occurrence of low-energy hip fractures over up to 7 years of follow up (Figure 2). A hip fracture leads to hospitalisation, and it is therefore comprehensively registered. A low-energy fracture was defined as a discharge diagnosis of hip fracture (ICD-10 codes S32.1, S32.3, S32.4, S72.0–S72.4, S72.7, and S72.8) without any other fracture code and without road-traffic accident codes as extrinsic factors. The codes of extrinsic factors such as “fall on the same level” and “fall on the snow or ice” were applied in the sensitivity analyses. The dates for death and institutionalisation were provided by the SII.

### Patient Characteristics

Patients’ age at the end of the year prior to the cohort entry, hospital catchment area of the place of the residence, and the calendar year of the cohort entry were recorded. We assessed prescription drug use (insulin and other blood glucose lowering drugs, imidazoline receptor agonists, diuretics, beta blockers, calcium channel blockers, angiotensin-converting enzyme inhibitors, angiotensin receptor blockers, hormone replacement therapy, inhaled corticosteroids, bisphosphonates, calcitonin, thyroxin, phenytoin, selective serotonin reuptake inhibitors) within the 365 days prior to the cohort entry and co-morbidities (coronary artery disease, rheumatoid arthritis, cardiac insufficiency, cardiac arrhythmias, epilepsy, parkinsonism), as captured by the SII registers, any time before the cohort entry.

### Statistical Analysis

We determined the incidence of hip fractures (per 1000 person-years) among the cohorts. The hazard ratio (HR) was estimated with Cox proportional-hazards regression. For confounder adjustment, we used the propensity score (PS) method. The propensity for adherence to statins was estimated separately in the statin versus hypertension cohort analysis and in the statin versus population cohort analysis by fitting a logistic regression model including the characteristics measured at cohort entry (see above). For the exposure-response analyses within the statin cohort, separate PS models were constructed for propensity for good versus moderate statin adherence and for good versus poor statin adherence, respectively. For other exposure-response analyses, several models were constructed modeling the PS for statin use in each case. When comparing the statin users with the population, the region of residence was excluded from the PS due to missing data (12% of the population cohort). In addition to the exposure group and the PS, the outcome models included those individual confounding factors that were the strongest predictors of the outcome (p<0.0001) [21]. When comparing the statin and hypertension cohorts, age, coronary heart disease, rheumatoid arthritis, use of antidiabetic drugs, and use of hormone replacement therapy were added in the model. In the statin versus population analyses, these were further supplemented by the use of diuretics, beta blockers, calcium channel blockers, and angiotensin-converting enzyme inhibitors or angiotensin receptor blockers.

The following exposure-response relationships were analysed by comparing: 1) the incidence in different groups of adherence to statins (<40%, 40–80%, ≥80% plus at least 3 purchases in each year) with the incidence in the adherent users of antihypertensive drugs and 2) with the incidence in the population cohort, and 3) the incidence across adherence groups within the statin cohort. For calculating P-values for trend, adherence to statins was defined as a continuous variable obtaining values from 0 (no statin exposure) to 3 (adherence to statins ≥80%).

In *post hoc* subgroup analyses, we first restricted the analyses to hip fractures caused by falls on the same level, or falls on snow or ice, by using the extrinsic factor codes provided in the Finnish Care Register. Second, we included those with any fracture within the 365 days prior to and during the exposure period to the study population. Finally, we examined the effect of various modifications of the PS on the HRs. First, use of antipsychotics, anxiolytics, hypnotics and sedatives, non-selective monoamine-reuptake inhibitors, monoamine oxidase type A inhibitors, or other antidepressants during the 365 days prior to the follow-up were added to the PS model. Second, in a separate sensitivity analyses, each of the following variables measured during the exposure period were included in the PS: alcohol-related hospitalisation, use of bisphosphonates acting on mevalonate pathway (pamidronic acid, alendronic acid, ibandronic acid, risedronic acid, zoledronic acid, risedronic acid in combination preparation with calcium, or risedronic acid with calcium and cholecalsiferol), and use of medication indicated in diabetes or hypertension.

The study size was based on *a priori* estimation. During a 10-year period among the population aged 50 years or older in Finland, the mean number of the first records with hip fracture diagnosis was 5564 per year in the early 2000’s [22]. In women, the number was 4050 per year (Sund, personal communication) yielding an incidence of 0.38% which we used as the incidence estimate for the unexposed. A risk ratio of 0.75 [4] with the two-sided significance level of 5% and 80% power was applied in calculations conducted by the Epi-Info software (http://wwwn.cdc.gov/epiinfo/). Aiming at the equal number of the exposed and unexposed, approximately 57600 person years would be needed in both groups.

We used SAS software (version 9.2; SAS Institute, Inc., Cary, NC, USA) for statistical analyses.

#### Ethical considerations

The SII, the National Data Protection Agency, and the National Institute for Health and Welfare, Helsinki, Finland approved the study protocol. There was no legal requirement for an ethics committee approval because only de-identified register data were used and the persons in the registers were not contacted (the Finnish legislation at: http://www.finlex.fi/fi/laki/ajantasa/1999/19990488). No written consent from patients was required either.

## Results

Of those 107 953 eligible women who initiated statin therapy in 1996–2001, 40 254 (37.3%) were included in the statin cohort (Figure 1). In the hypertension cohort, 41 610 (34.1%) out of 122 114 were included. As there were no exclusions based on adherence, 62 585 (90.2%) out of 69 705 eligible women were included in the population cohort. The cohorts differed in terms of age and co-morbidity distributions (Table 1). In the hypertension cohort, the distribution of the cohort entry year differed from that in the other two cohorts. The statin cohort was the oldest, mean age (SD) being 62.4 (7.5) years, with the highest prevalence of co-morbidities and use of various drugs at cohort entry. The population cohort was the youngest, 56.5 (8.7) years, with the lowest prevalence of co-morbidities.

**Table 1:** Selected characteristics of the persons in the study cohorts.

	StatinNo (%)(n = 40254)		HypertensionNo (%)(n = 41610)		PopulationNo (%)(n = 62585)	
**Age at cohort entry**						
45–55	8428	(20.94)	15997	(38.45)	33682	(53.82)
56–65	16128	(40.07)	10655	(25.61)	16556	(26.45)
66–75	15698	(39.00)	14958	(35.95)	12347	(19.73)
**Age, years, mean (SD)**	62.4	(7.5)	60.2	(9.5)	56.5	(8.7)
**Cohort entry**						
1996	4066	(10.10)	7694	(18.49)	6917	(11.05)
1997	5013	(12.45)	7018	(16.87)	8087	(12.92)
1998	5986	(14.87)	6758	(16.24)	9274	(14.82)
1999	7737	(19.22)	6650	(15.98)	11758	(18.79)
2000	9116	(22.65)	6650	(15.98)	13464	(21.51)
2001	8336	(20.71)	6840	(16.44)	13085	(20.91)
**365 days prior to the cohort entry use of**						
Beta blockers	16299	(40.49)	NA		6914	(11.05)
Hormone replacement therapy	12674	(31.49)	12172	(29.25)	15354	(24.53)
Diuretics	9067	(22.52)	7939	(19.08)	4596	(7.34)
Thiazides (alone or in combination preparations)	8887	(22.08)	6018	(14.46)	4457	(7.12)
Angiotensin-converting enzyme inhibitors or angiontensin receptor blockers	8749	(21.73)	NA		3766	(6.02)
Calcium channel blockers	7478	(18.58)	NA		2893	(4.62)
Diabetes drugs	5111	(12.70)	1830	(4.40)	1115	(1.78)
Inhaled corticosteroids	2917	(7.25)	2514	(6.04)	3766	(6.02)
Bisphosphonates acting through mevalonate pathway*	468	(1.16)	386	(0.93)	352	(0.56)
Other bisphosphonates†	91	(0.23)	89	(0.21)	85	(0.14)
**Any time prior to the cohort entry evidence of** ‡						
Coronary artery disease	8072	(20.05)	1588	(3.82)	1295	(2.07)
Rheumatoid arthritis	1262	(3.14)	1797	(4.32)	1660	(2.65)
Cardiac insufficiency	1659	(4.12)	1589	(3.82)	689	(1.10)
Cardiac arrhythmias	923	(2.29)	687	(1.65)	452	(0.72)
Epilepsy	450	(1.12)	435	(1.05)	513	(0.82)
Parkinsonism	106	(0.26)	163	(0.39)	205	(0.33)

* Pamidronic acid, alendronic acid, ibandronic acid, risedronic acid, zoledronic acid, risedronic acid in combination preparation with calcium, or risedronic acid with calcium and cholecalsiferol.

† Etidronic acid, clodronic acid, tiludronic acid, or etidronic acid with calcium.

‡ As indicated in the Special Reimbursement Register of the Social Insurance Institution in Finland.

In the statin cohort, 199 women (0.50%) sustained hip fracture during a mean of 3.36 years of follow-up, the respective figures in the hypertension and population cohorts were 312 (0.75%, 3.78 years) and 212 (0.34%, 3.46 years) (Table 2). The incidence rate was the highest in the hypertension cohort (2.0 hip fractures per 1000 person-years), followed by the statin (1.5) and the population cohorts (1.0). Of the statin cohort, 1 397 (3.45%) persons died during the follow-up. The respective figures in the hypertension and population cohorts were 2 256 (5.42%) and 1 691 (2.70%).

**Table 2:** Patient follow-up, low-energy hip fracture, and incidence rates in the study cohorts.

	Statin (n = 40254)	Hypertension (n = 41610)	Population (n = 62585)
**Follow-up time, years (mean, SD)**	3.36 (1.64)	3.78 (1.86)	3.46 (1.66)
**Events**	199	312	212
**Total follow-up time, person-years**	135329.69	157090.10	216329.69
**Incidence rate per 1000 person-years**	1.5	2.0	1.0

SD = standard deviation.

When adjusting for the propensity score and individual variables strongly predicting the outcome, good adherence to statins for five years was associated with a 29% decreased risk (HR 0.71; 95% CI 0.58–0.86) of a low-energy hip fracture when comparing with good adherence to antihypertensive drugs (Table 3). The association was of the same magnitude when comparing the statin users with the population cohort, HR being 0.69 (0.55–0.87).

**Table 3:** Risk of low-energy hip fracture in adherent users of statins compared with adherent users of antihypertensive drugs and with randomly selected population cohort.

	Statin versus hypertension cohort(n = 81856*) HR (95% CI)	Statin versus population cohort(n = 102839) HR (95% CI)
**Crude**	0.75 (0.63–0.90)*	1.51 (1.24–1.83)
**Adjusted for age and year of cohort entry**	0.76 (0.63–0.91)*	0.93 (0.76–1.13)
**Adjusted for propensity score and variables strongly** **associated with** **the outcome** †,‡	0.71 (0.58–0.86)*	0.69 (0.55–0.87)

* 8 persons missing data on region of residence excluded.

HR = Hazard ratio.

† Age, coronary heart disease, rheumatoid arthritis, use of anti-diabetics, and hormone replacement therapy at cohort entry in the statin versus hypertension cohort analyses.

‡ Variables mentioned above plus diuretics, beta blockers, calcium channel blockers, and angiotensinconverting enzyme inhibitors or angiotensin receptor blockers at cohort entry in the statin versus population cohort analyses.

### Exposure-response Relationships

In order to analyse the exposure-response relationship, hip fracture incidence rates in women with poor (<40%), moderate (40–80%) and good (≥80% plus at least 3 purchases per year) adherence to statins, respectively, were compared with those in women with good adherence to the antihypertensive drugs. HRs decreased from 0.89 (0.70–1.12) through 0.73 (0.58–0.91) to 0.71 (0.58–0.86) (*P* for trend 0.0002, Table 4). The respective phenomenon was found in the statin versus population analyses; HRs decreased from 0.87 (0.68–1.10) through 0.71 (0.51–1.00) to 0.69 (0.55–0.87) (*P* for trend 0.0005, Table 4).

**Table 4:** Exposure-response relationships.

	CrudeHR (95% CI)	Adjusted for age and yearof cohort entry HR (95% CI)	Adjusted for propensity score and variables stronglyassociated with the outcome HR(95% CI)
**Statin vs hypertension cohort**			
Poor adherence* to statins vs goodadherence to hypertension drugs (n = 62885)	0.76 (0.61–0.95)	0.89 (0.72–1.12)	0.89 (0.70–1.12)
Moderate adherence† to statins vs good adherence to hypertension drugs (n = 70091)	0.65 (0.53–0.80)	0.74 (0.60–0.91)	0.73 (0.58–0.91)
Good adherence‡ to statins vs goodadherence to hypertension drugs (n = 81864)	0.75 (0.63–0.90)**¥**	0.76 (0.63–0.91)**¥**	0.71 (0.58–0.86) **¥**
**Statin vs population cohort**			
Poor adherence* to statins vs population (n = 83887)	1.49 (1.18–1.89)	1.06 (0.83–1.34)	0.87 (0.68–1.10)
Moderate adherence† to statins vs population (n = 91072)	1.30 (1.04–1.63)	0.90 (0.72–1.12)	0.71 (0.51–1.00)
Good adherence‡ to statins vs population (n = 102839)	1.51 (1.24–1.83)**¥**	0.93 (0.76–1.13)**¥**	0.69 (0.55–0.87) **¥**
**Within the statin cohort**			
Persons with good‡ vs poor* adherence to statins(n = 61566)	0.99 (0.79–1.26)	0.85 (0.67–1.08)	0.82 (0.65–1.03)
Persons with good‡ vs moderate†adherence to statins(n = 68741)	1.15 (0.92–1.44)	1.03 (0.82–1.28)	1.01 (0.80–1.26)

Risk for low-energy hip fracture.

HR = Hazard ratio.

* Poor adherence = prescribed days statins covered <40% of the 5-year exposure period.

† Moderate adherence = prescribed days statins covered ≥40% and <80% of the 5-year exposure period.

‡ Good adherence = prescribed days statins/hypertension drugs covered ≥80% of the 5-year exposure period and at least 3 purchased statin/hypertension drug prescriptions in each year.

¥ Hazard ratios presented also in Table 3.

Persons missing data on region of residence excluded in all analyses.

Within the statin cohort, hip fracture risk tended to be lower in the women with good adherence in comparison with the poor adherence group (HR 0.82; 0.65–1.03) but no difference was found between the moderate and good adherence groups (HR 1.01; 0.80–1.26) (Table 4).

### Subgroup Analyses

The association between adherent use of statins and hip fracture did not change when the outcome was defined as the fracture caused by fall on the same level, or on the snow or ice (Table 5). Using the above end point definition, the HR was 0.69 (0.56–0.86) in the statin versus hypertension cohort comparison and 0.68 (0.52–0.88) in the statin versus population comparison.

**Table 5:** Subgroup analysis.

	Statin cohort versushypertension cohort HR(95% CI)	Statin cohort versus population cohortHR (95% CI)
**Restricted to registered falls on the same level,** **or falls on snow or ice**	0.69 (0.56–0.86)*(n = 81856, 413 events of the total 511)	0.68 (0.52–0.88)(n = 102839, 327 events of the total 411)
**Any fracture in the 365 days prior to the** **exposure time examined in the propensity score and no exclusion based on the fractures**	0.68 (0.57–0.80)*(n = 86259)	0.72 (0.59–0.88)(n = 107552)
**Any fracture in the 365 days prior to and during** **the exposure time ignored (no exclusion** **based on fractures)**	0.67 (0.57–0.79)*(n = 86259)	0.73 (0.60–0.89)(n = 107552)

Risk for low-energy hip fractures.

* Persons missing data on region of residence excluded.

HR = Hazard ratio.

Hazard ratios were adjusted for propensity score and variables strongly associated with the outcome.

### Sensitivity Analyses

In sensitivity analyses, various variables measured during the exposure period were added to the PS but these additions did not affect the HRs (change in the estimate <10%) in the outcome models (Table 6).

**Table 6:** Sensitivity analyses.

	Statin cohort versus hypertensioncohort n = 81864* HR (95% CI)	Statin cohort versus populationCohort n = 102839 HR (95% CI)
**Use of antipsychotics, anxiolytics, hypnotics and sedatives,** **non-selective monoamine-reuptake inhibitors,** **monoamine oxidase type A inhibitors, or other antidepressants** **during the 365 days prior to the follow-up added**	0.70 (0.58–0.85)	0.68 (0.53–0.85)
**Alcohol-related hospitalisation during the exposure** **period added**	0.71 (0.58–0.86)	0.69 (0.54–0.88)
**Use of bisphosphonates (mevalonate pathway)** † **during** **exposure period added**	0.71 (0.58–0.86)	0.69 (0.54–0.87)
**Use of medication indicated in diabetes or hypertension** ‡ **during the exposure period added**	0.71 (0.58–0.86)	0.64 (0.49–0.83)

Risk for low-energy hip fracture. Various modifications in the propensity score examined.

HR = Hazard ratio.

Hazard ratios were adjusted for propensity score and variables strongly associated with the outcome.

* Persons missing data on region of residence excluded.

† Pamidronic acid, alendronic acid, ibandronic acid, risedronic acid, zoledronic acid, risedronic acid in combination preparation with calcium, or risedronic acid with calcium and cholecalsiferol.

‡ Insulin and other blood glucose lowering drugs, diuretics, beta blockers, calcium channel blockers or angiotensin-converting enzyme inhibitors and angiotensin receptor blockers.

Distribution of the variables used in the propensity score examinations are presented in the Table S1.

## Discussion

When women aged 50–80 years who had been adherent to statins for 5 years were compared with women adherent to antihypertensive drugs or with a cohort randomly selected from the population, statin users were at a 30% decreased risk of hip fracture over a mean of 3.57 years of follow-up. Furthermore, when women with poor, moderate, and good adherence to statins were compared to those with good adherence to antihypertensive drugs or to the population cohort, the protective effect associated with statin use increased with the increasing level of adherence.

Within the statin cohort, no difference in the risk of hip fractures was found between women with moderate (40–80%) and good adherence (≥80% and at least three prescriptions per year) while women with poor adherence (<40%) had a non-significant 20% increase in risk compared with those having good adherence. The finding, together with the results of other exposure-response analyses, may indicate that even less intensive statin exposure may be sufficient for improving bone health. When the analysis was adjusted for both the PS and the use of drugs indicated in diabetes or hypertension (including diuretics) during the follow-up the results of the statin/hypertension analysis did not change but the HR of the statin/population decreased to 0.64 (0.49–0.83) (Table 6).The baseline characteristics of the women with poor and good adherence differed; the mean age was higher and the frequencies of cardiovascular medications and coronary artery disease were higher in the women with good adherence (Table S2). Consequently, better general health in the group with good adherence cannot explain the results of the comparisons within the statin group.

The results of our study with a long exposure duration are concordant with the results from the post-hoc analyses of the LIPID study in women [5] and with the results of previous observational studies with heterogeneous exposure definitions in women [4]. However, the associations found in our analyses are not as strong as those published more recently. In a Danish population-based case-control study, 5-year statin adherence of at least 58% (defined as good) was associated with a 43% reduction (OR 0.57; 95% CI 0.39–0.84) in hip fracture risk in women [23]. In the same study, good statin adherence was associated with a 89% reduction (OR 0.11; 95% CI 0.02–0.66) in hip fracture risk in men aged 65 or younger. Furthermore, in a case-control study from the USA, already a 3-month statin use was associated with a 67% reduction (OR 0.23; 95% CI 0.09–0.57) in the hip fracture risk in elderly women using hormone replacement therapy [14]. These estimates suggest that benefits of statin use would exceed the achievements of conventional osteoporosis therapy [24] which does not seem biologically plausible. Inadequate adjustment for health seeking behavior and other types of residual confounding may partly explain the above exceptional findings.

The proposed protective effect of statins in bone may be explained by their biochemical effects on osteoblasts and osteoclasts. By interfering in the mevalonate pathway, statins suppress the synthesis of isoprenoids, such as farnesyl- and geranylgeranyl pyrophosphate [2], which mimics the effect of nitrogen-containing bisphosphonates inhibiting the same pathway at the level of farnesyl pyrophosphate synthase [25]. Simvastatin inhibits osteoclast differentiation [26], and both lipophilic [27] and hydrophilic statins [28] induce osteoblast differentiation. In bone of ovariectomized rats, simvastatin enhances the production of osteogenic proteins [29] and induces oestrogen receptor-alpha expression [30]. Since oestrogens slow down bone resorption and increase bone mineral density [31], induction of oestrogen receptor expression by statins, if functional also in postmenopausal women, could attenuate the effect of decrease in oestrogen levels or potentiate the effect of hormone replacement therapy on bone. Existence of a specific pharmacological effect is further supported by the finding that the fatty acid composition as such has no significant role in bone health [32].

### Strengths

The strengths of our study include the comprehensive ascertainment of the outcome events, a long exposure period, *a priori* power analysis, the adjustment for several confounders, the observed exposure-response relationship, and the use of two different comparison groups. We chose the adherent hypertension cohort as a primary comparison group in order to control for health seeking behaviour and, additionally, for some covariates, such as body mass index (BMI), not available in the registers. In postmenopausal women, total body fat, clinically considered in BMI, is positively associated with the BMD [33], a predictor of fracture [34]. High BMI, in turn, is associated both with hypertension and hypercholesterolemia [35].

### Limitations

The present study has weaknesses. Our definition of exposure, adherence to statin therapy, is two-dimensional; we cannot differentiate whether the associations or exposure-response relationships are due to accumulating dose or due to adherent behaviour, or both. Adherence to statin therapy may represent health seeking behaviour consisting of numerous elements, including use of preventive health services [36]. By choosing the adherent users of antihypertensive drug as the main comparator group we aimed to create groups similar in that respect. While doing that, however, we may have introduced selection bias. Women on antihypertensive drugs who initiated statin therapy during 1996–2006 were selected to the statin cohort and excluded from the hypertension cohort, the future statin initiation modifying the selection. Consequently, frail women, women with several co-morbidities, with less interest in health seeking, or with worse life-time prognosis, i.e. those with high risk for hip fracture may have been overrepresented in the hypertension cohort. In the statin versus population comparison, we primarily hypothesised to find a protective association due to health seeking behaviour of statin users [36]. Specially, in this analysis, uncontrolled health seeking behavior may have biased the effect estimates. We chose to study the incidence rate of low-energy hip fracture as hip fracture has been considered as an index fracture of osteoporosis. Hip fractures, however, result from falls that are associated with functional capacity, disability, and general health that we could not control for. We could not control for use of vitamin D and calcium supplements, dietary intake of them, physical activity, genetic factors, or frailty. Even though we controlled for oestrogen use, we did not take into account the duration or intensity of exposure. We did not study use of non-statin lipid lowering drugs as their consumption in Finland decreased during the study years from 12.8% in 1996 to 1.9% in 2006 of the total lipid lowering drug consumption measured as Defined Daily Doses/1000 inhabitants/day [37], [38].

### Implications

Hip fracture can be considered as a burden to health care systems worldwide, although the overall rate of hip fractures varies across countries due to both genetic and environmental factors [39]. Contemporary pharmacotherapy, such as bisphosphonates, is rather ineffective in prevention of low-energy, osteoporotic fractures [40], [41], [42]. For example, the NNT for hip fracture prevention over 3 years in an osteoporotic subset of the population ranged from 48 (strontium ranelate) to 91 (bisphosphonates) [43]. Our results can be translated into a naivé number needed to treat (NNT) [44]; 628 postmenopausal women without prior hospitalisations for fractures need to use statins adherently for at least 5 years in order to prevent one future low-energy hip fracture over 3 years of follow-up. The respective NNT for 5 years of follow-up is 346. These estimates apply to a female population with cardiovascular risk factors (but without data on BMD), i.e. women using medication for hypercholesterolemia or for hypertension. In further studies, impact of time-varying confounding factors and the impact of various exposure durations on fracture risk are worth exploring. Especially, the cumulative dose may be relevant as in animal studies the doses have been ten times that used in routine clinical practice [1].

### Conclusions

In conclusion, our large population-based study suggests that long-term exposure to statins is associated with a reduced risk of low-energy hip fracture in women aged 50–80 years without prior hospitalised fractures. Although the absolute benefit is small, some extra benefit in bone health may be achieved when statins are used for their main indication.

## Supporting Information

Table S1 **Selected characteristics of the persons in the study cohorts.**
(PDF)

Table S2 **Selected characteristics of the persons within the statin cohort.**
(PDF)
