# Supplementary material for: Statins and Hip Fracture Prevention – A Population Based Cohort Study in Women
Source: PLoS One. 2012 Oct 29;7(10):e48095. doi: 10.1371/journal.pone.0048095 (PMC3483280; doi:10.1371/journal.pone.0048095)
Supplement: Table S1 — Selected characteristics of the persons in the study cohorts. (PDF) [file pone.0048095.s001.pdf]

Table S1. Selected characteristics of the persons within the statin cohort.

	Persons with poor* adherence N (%)		Persons with good† adherence N (%)	
**Age at cohort entry**				
45-55	9940	(29.10)	8428	(20.94)
56-65	13128	(38.44)	16128	(40.07)
66-75	11085	(32.46)	15698	(39.00)
**Year of the cohort entry**				
1996	3787	(11.09)	4066	(10.1)
1997	4606	(13.49)	5013	(12.45)
1998	4874	(14.27)	5986	(14.87)
1999	6315	(18.49)	7737	(19.22)
2000	7395	(21.65)	9116	(22.65)
2001	7176	(21.01)	8336	(20.71)
**365 days prior to the cohort entry use of**				
Beta blocking agents	12343	(36.14)	16299	(40.49)
Hormone replacement therapy	10627	(31.12)	12674	(31.49)
Diuretics	6813	(19.95)	9067	(22.52)
Thiazides (alone or in combination preparations)	6965	(20.39)	8887	(22.08)
Angiotensin-converting enzyme inhibitors or angiotensin receptor blockers	6756	(19.78)	8749	(21.73)
Calcium channel blockers	5558	(16.27)	7478	(18.58)
Diabetes drug	3439	(10.07)	5111	(12.7)
Inhaled corticosteroids	2393	(7.01)	2917	(7.25)
Bisphosphonates acting through mevalonate bathway‡	344	(1.01)	468	(1.16)
**Any time prior to the cohort entry evidence of¥**				
Coronary artery disease	4816	(14.1)	8072	(20.05)
Rheumatoid arthritis	1152	(3.37)	1262	(3.14)
Cardiac insufficiency	1108	(3.24)	1659	(4.12)
Cardiac arrhythmias	669	(1.96)	923	(2.29)
Epilepsy	343	(1)	450	(1.12)
Parkinsonism	73	(0.21)	106	(0.26)

*Poor adherence=prescribed days statins covered <40% of the 5-year exposure period.
†Good adherence=prescribed days statins covered ≥80% of the 5-year exposure period and at least 3 purchased statin prescriptions in each year.
‡Pamidronic acid, alendronic acid, ibandronic acid, risedronic acid, zoledronic acid, risedronic acid in combination preparation with calcium, or risedronic acid with calcium and cholecalsiferol
¥As indicated in the Special Reimbursement Register of the Social Insurance Institution in Finland
