# Supplementary material for: Statins and Hip Fracture Prevention – A Population Based Cohort Study in Women
Source: PLoS One. 2012 Oct 29;7(10):e48095. doi: 10.1371/journal.pone.0048095 (PMC3483280; doi:10.1371/journal.pone.0048095)
Supplement: Table S2 — Selected characteristics of the persons within the statin cohort. (PDF) [file pone.0048095.s002.pdf]

Table S2. Characteristics of the persons in the study cohorts.

	Statin No (%) (n=40254)		Hypertension No (%) (n=41610)		Population No (%) (n=62585)	
**During the exposure period**						
Alcohol-related hospitalisation	4	(0.01)	11	(0.03)	16	(0.03)
Use of bisphosphonates (mevalonate bathway)*	2226	(5.53)	2004	(4.82)	1833	(2.93)
Insulin and other blood glucose lowering drugs	7393	(18.37)	4021	(9.66)	2239	(3.58)
Diuretics	10666	(26.50)	12832	(30.84)	5632	(9.00)
Beta blocking agents	21815	(54.19)	23684	(56.92)	9765	(15.60)
Calcium channel blockers	9751	(24.22)	11165	(26.83)	4260	(6.81)
Angiotensin-converting enzyme inhibitors or angiontensin receptor blockers	15383	(38.17)	20325	(48.85)	7736	(12.36)
**During the 365 days prior to the follow-up**						
Use of antipsychotics, anxiolytics, hypnotics, sedatives, non-selective monoamine-reuptake inhibitors, monoamine oxidase type A inhibitors, or other antidepressants	9770	(24.27)	9034	(21.71)	8390	(13.41)

*Pamidronic acid, alendronic acid, ibandronic acid, risedronic acid, zoledronic acid, risendronic acid in combination preparation with calcium, or risedronic acid with calcium and cholecalsiferol
